# Protective effect of a fecal incontinence management system against bacteremia for out-of-hospital cardiac arrest patients undergoing extracorporeal membrane oxygenation

**DOI:** 10.1186/cc14182

**Published:** 2015-03-16

**Authors:** S Kikuta, R Miki, S Ishihara, S Nakayama

**Affiliations:** 1 Hyogo Emergency Medical Center, Chuo, Kobe, Hyogo, Japan

## Introduction

Recently, extracorporeal cardiopulmonary resuscitation (ECPR) has become a common measure against cardiopulmonary arrest. In cases with ECPR, we usually insert cannulae for extracorporeal membrane oxygenation (ECMO) via the femoral artery and vein. However, the cannulation site is often contaminated by feces due to incontinence. Moreover, patients tend to be compromised by hypothermia due to the target temperature management, so we often experience central line-associated bloodstream infection of patients undergoing ECMO. We investigated the protective effect of a fecal incontinence management system (FMS) against bacteremia in patients undergoing ECMO.

## Methods

We studied 41 consecutive patients undergoing ECMO for out-of-hospital cardiac arrest (OHCA) between April 2010 and May 2014. Patients were divided into two groups according to the use or no use of FMS (Flexi-Seal™). Patients who died within 48 hours or from whom cannulae for ECMO were removed within 48 hours were excluded. Patients' characteristics, underlying disease, target temperature management, prophylactic antibiotic use and incidence of bacteremia during admission were recorded and analyzed retrospectively.

## Results

Among 41 patients, 15 (37%) underwent FMS. There was no difference in age, sex, underlying disease, target temperature management, and prophylactic antibiotic use between two groups. Mean duration of ECMO was 4 days in both groups. The incidence of bacteremia was none in the group with FMS and five (19%) in the group without FMS. Within five cases of bacteremia, three were caused by enterobacterium.

## Conclusion

FMS may be protective against bacteremia for OHCA patients undergoing ECMO.

